# The Knowledge Translation Status in Selected Eastern-Mediterranean Universities and Research Institutes

**DOI:** 10.1371/journal.pone.0103732

**Published:** 2014-09-08

**Authors:** Katayoun Maleki, Randah R. Hamadeh, Jaleh Gholami, Ahmed Mandil, Saima Hamid, Zahid Ahmad Butt, Abdulaziz Bin Saeed, Dalia Y. M. El Kheir, Mohammed Saleem, Sahar Maqsoud, Najibullah Safi, Ban A. Abdul-Majeed, Reza Majdzadeh

**Affiliations:** 1 Knowledge Utilization Research Center, TUMS, Tehran, Iran; 2 College of Medicine and Medical Sciences, AGU, Manama, Kingdom of Bahrain; 3 College of Medicine, KSU, Riyadh, KSA; 4 Health Services Academy, MoH, Islamabad, Pakistan; 5 Department of Family & Community Medicine, KSU, Riyadh, KSA; 6 Faculty of Medicine, University of Khartoum, Khartoum, Sudan; 7 High Institute of Public Health, Alexandria University, Alexandria, Egypt; 8 Preventive Affairs Department, Alexandria University Students' Clinic and Hospital, Alexandria, Egypt; 9 WHO Country Office, Kabul, Afghanistan; 10 College of Medicine, Al-Nahrain University, Baghdad, Iraq; Newcastle University, United Kingdom

## Abstract

**Background:**

A serious worldwide effort to strengthen research based knowledge translation (KT) has begun in recent years and some countries, particularly developed ones, are trying to incorporate KT in their health and health research systems. Keeping in mind the recent economic depression and the need to perform more efficient research, we aimed to assess and compare the KT status of selected health research institutes in the Eastern Mediterranean Regions' countries, and to identify their strengths and weaknesses in the field.

**Methods:**

After finding the focal points that would steer the focus group discussions (FGDs) and help complete the ‘Self Assessment Tool for Research Institutes’ (SATORI) tool, each focal point held two FGDs in which researchers, research authorities and other individuals specified in detail further in the study were held. The scores obtained by each institute were evaluated quantitatively, and the transcriptions were analyzed qualitatively with OpenCode software.

**Results:**

For ease of analysis the 50 items of the SATORI were classified into 7 main domains: ‘priority setting’, ‘research quality and timeliness’, ‘researchers’ KT capacities', ‘facilities and pre-requisites of KT’, ‘processes and regulations supporting KT’, ‘interaction with research users’, and ‘promoting and evaluating the use of knowledge’. Based on the scoring system, the strongest domain was ‘research quality and timeliness’. ‘Priority setting’ was the weakest domain of all. The remaining domains were more or less equal in strength and were not in a favorable state. The qualitative findings confirmed the quantitative findings.

**Conclusions:**

The main problem, it seems, is that a KT climate does not exist in the region. And despite the difference in the contexts, there are many similarities in the region's institutes included in this study. Collaborative efforts can play a role in creating this climate by steering countries towards KT and suggesting regional strategic directions according to their needs.

## Introduction

“Knowledge translation (KT) is defined as a dynamic and iterative process that includes the synthesis, dissemination, exchange and ethically sound application of knowledge to improve health, provide more effective health services and products, and strengthen the health care system” [1]. In today's world, KT is considered as an important solution for promoting community health, achieving up-to-date and quality healthcare, accelerating the conversion of research into action and bridging the gap between knowledge and decision-making. In spite of the great deal of financing done in health research and the fact that many of these research results are available to decision makers, we are still witnessing the gap in evidence based decision making (EBDM) [2]. Around the world KT is increasingly being recognized as an integral element of health care, where research can be best utilized to the benefit of the community [3]. In fact, EBDM is being given more importance than ever in the health system [4]. Clinical and public health guidelines, patient decision aids, and policy briefs are live examples of evidence being applied to clinical or public practice in the health arena. The recent economic depression in the world gives countries all the more incentive to take cost-effectiveness into account in their expenses, especially when it comes to research [5].

KT occurs in a complex system of interactions among knowledge producers and users; and in addition to the efforts made by decision makers to search for and utilize evidence, research producers should also try to deliver and communicate their findings to decision makers [6]. A narrative review of literature shows that at national and organizational level, collective models and not individual models affect knowledge exchange. In fact, three dimensions of context, i.e. politics, economics, and social structure affect it [7], [8]. Previously, focus has been laid on climate and production as well [6]. Furthermore, earlier studies have shown that in addition to factors such as relations between researchers and policy makers, stewardship is the most influential factor in KT [9]. Studies show that in addition to individual factors, organizational factors also influence KT activities in research organizations. The pre-requisite of such activities is the existence of research systems that have been modified by researchers active in the field of KT [10].

A serious worldwide effort to strengthen research-based KT has begun since 2004 and some countries, particularly developed ones, are trying to incorporate KT in their health and health research systems [11], [12]. In a summit held in 2008 for strengthening primary health care, the Eastern Mediterranean Region (EMR) countries proposed KT strengthening as one of their commitments in the 30^th^ anniversary of the Alma-Ata Declaration [13]. In the Fifty-eighth session held by the ‘Regional Committee for the Eastern Mediterranean’ in 2011 five goals were delineated. One of which was to “Improve the access of governments and decision-makers to research evidence necessary to inform health policy and practice” [14].

Participants in the session were asked to take appropriate and necessary measures. Within the 22 countries of the EMR however, the status quo is otherwise, and KT is yet rather unknown and in its prime; only a few countries have been working on the subject in recent years [15], [16]. Moreover, most of these countries are in an unfavorable state when it comes to health and health research indicators; many lacking efficient research system structures [17].

The objectives of this study were to assess and compare the KT status of selected medical universities and health research institutes in EMR countries and to identify their strengths and weaknesses in the field.

## Methods

In 2009 a workshop organized by the World Health Organization's EMR Office (WHO/EMRO) titled "Use of Knowledge and Research Evidence for Improved Health Policy" was held [18]. Thirty five participants from 15 EMR countries participated in this workshop. Participants were both from the Ministries of Health and academic institutes. The research team emailed the participants and asked them to introduce or recommend focal points ‘or’ introduce researchers or people who would be suitable for this role. Unfortunately, no responses to our mailing were received.

The ‘focal point’ we had in mind was an academic member or university authority who was familiar with research methodology and who would be able to organize and facilitate the FGDs that were set up for data collection. The focal point was also required to keep in touch with the PI and co-investigator and to send all the completed files at the end of data collection.

At the same time, we extensively searched for medical schools and universities in the region and selected the top three institutes from a complete list of the entire region's medical schools and universities in each country. The selection was based on having had more than 100 publications in PubMed. We then contacted the WHO/EMRO and requested forwarding our message to such institutes. The office kindly sent the official letter to invite 12 institutes from different EMR countries to participate in the project. Only one institute responded to this invitation and announced its willingness to participate in the study.

When the two above methods failed, we began searching for focal points by using the keywords ‘*country name, community medicine, article, email*’, to identify emails of researchers currently active in community medicine. The EMR countries were searched several times and a total of 81 researchers were identified and contacted. At the most, each researcher was invited to participate in the study (via email) three times. Through this method, three researchers accepted to cooperate as focal points. The other four focal points were approached face-to-face by the principal investigator (PI). Eventually, apart from Iran, only 8 out of 20 medical universities & health research institutes participated in the study. One of these institute's data lacked sound quality, and was therefore not included in the final data analysis.

The EMR countries of the participating institutes have different demographic and health statistics, listed in table 1. The WHO Regional Office for the Eastern Mediterranean has classified these countries on the basis of their health system performance and level of health expenditure into three groups, illustrated in table 1 [19]. Practically speaking, group 1 countries are the Persian Gulf's oil-rich countries that have a high per capita income. Group 3 countries are those that have a low income, and group 2 countries lie between the latter two groups.

**Table 1:** Health and social statistical indicators of the EMR countries.

Country	Population 2012†	GDP per capita (US $)†	General government expenditure on health as % of total government expenditure	Life expectancy at birth†		Income levels†	Human Development Index (HDI) ‡
				Value	Year		
**Afghanistan** ×	26,500^a^	584	3.3	62.0*	2010	Low income	0.374
**Bahrain**	1,195	19512	9.2	75.3	2011	High income	0.796
**Djibouti**	865^a^	1336	14.1	52.9	2011	Low income	0.445
**Egypt**	82,541	2801	6.9	70.4	2012	Middle income	0.662
**Iran**	75,150	5819	10.1	72.1	2011	Middle income	0.742
**Iraq**	34,207	3993	10.2	72.7	2010	Middle income	0.590
**Jordan**	6,388	4655	17.6	73.0	2011	Middle income	0.700
**Kuwait**	3,632	56426	5.9	77.7	2009	High income	0.790
**Lebanon**	4,104	9904	5.8	81.5	2009	Middle income	0.739
**Libya**	5,922	9063	7.9	72.3	2009	Middle income	0.769
**Morocco**	3,2597	3082	6.5	74.8	2010	Middle income	0.591
**Oman**	3,623	25536	4.9	76.2	2012	High income	0.731
**Pakistan**	177,100^a^	1184	3.6	66.0	2011	Low income	0.515
**Palestine**	4,357	1697	10.0	72.7	2012	Middle income	0.670
**Qatar**	1,733^a^	92789	5.8	78.2	2010	High income	0.834
**Saudi Arabia**	2,9196	20540	6.8	73.8	2011	High income	0.782
**Somalia**	8,698^b^	284	NA	50.0	2010	Low income	NA
**Sudan**	3,3976	1234	10.6	59.8	2008	Low income	0.414
**Syria**	2,1639	2702	5.6	73.1	2009	Middle income	0.648
**Tunisia**	1,0674	4331	10.8	74.9	2011	Middle income	0.712
**United Arab Emirates**	8,264^b^	49005	8.8	77.4	2008	High income	0.818
**Yemen**	22,879^b^	1617	4.3	62.0	2010	Low income	0.458

a = 2011 ^b^ = 2010

† Demographic, Social and Health Indicators for Countries of the Eastern Mediterranean 2013

‡ Human Development Report 2013: http://hdr.undp.org/en/content/human-development-report-2013

*Afghanistan Mortality Survey 2010

× The countries that have been underlined are those that have participated in our study.

To allow a visual comparison of participating and non-participating countries, all 22 EMR countries are shown in the table.

### Data collection

The ‘Self Assessment Tool for Research Institutes’ (SATORI) was used to assess the status of KT in the EMR institutes. SATORI, meaning “understanding” is a Japanese Buddhist term for enlightenment. This tool's reliability and validity have been evaluated in an earlier study, and it has been used for evaluation in Iran's medical universities [20], [21]. The tool can be used by research authorities and researchers of any research institute (be it university, faculty, private or public research center or institute) to identify the barriers to KT ‘push’ efforts within the research organization and suggest appropriate solutions to improve the status quo. Each of the tool's 50 items addresses one of the aspects of KT. The items are scored upon consensus in the focus group discussion (FGD), using a five-point Likert scale. 1 means that “the situation is quite unfavorable and/or there is a dire need for intervention” and 5 means “the situation is acceptable and there is no need for intervention”. The institutes' researchers, research authorities and stakeholders should be present in the FGDs.

Both the tool and the protocol sent to the focal points contained a detailed guide on how to complete the tool. Moreover, these steps were fully explained to the focal points in person or on the phone. They were also told to inform the PI and/or the co-investigator of their FGD timings. The co-investigator was on-call during those times to answer any possible questions raised during the FGDs.

The focal points were asked to hold at least two FGDs, in which (in addition to the focal point) the following would be present: deputy and/or director of research affairs, two researchers (at least one professor, one associate professor, one of whom was a lady) and three stakeholders from research utilizing organizations, especially the Ministry of Health (MoH).

Half the focal points were female and the other half were male. They held either MD or PhD degrees and were mostly heads of departments in their own institutes. On the whole, 15 FGD sessions were held. Each session was about two hours long. The participants of each FGD differed from each other (with the exception of the focal point). A minimum of 6 and a maximum of 8 participants were present in each FGD (mean number of participants = 7). The group discussions took place at the focal points' or participants' workplaces (mainly the institutes). The discussions were audio-recorded upon acquiring verbal consent from participants; transcriptions were prepared, and subsequently translated into English (if originally carried out in the local language which was predominantly Arabic). The tools were completed upon consensus in each FGD. One tool was completed in each FGD, which was sent along with the transcriptions/translations via post or email to the co-investigator for quality control and analysis. Data collection was done in 2011.

### Data management

The tool's 50 items are minimally required to clarify the institutes' weaknesses and strengths in the field of KT. They were classified into seven main domains, namely: ‘priority setting’, ‘promoting and evaluating the use of knowledge’, ‘researchers’ KT capacities’, ‘processes and regulations supporting KT’, ‘facilities and pre-requisites of KT’, ‘interaction with research users’, and ‘research quality and timeliness’. These domains have been previously extracted by the authors in a national study, using the same tool. The research team believes that this categorization helps correctly identify existing KT barriers and shortcomings [21].

Quantitative and qualitative analyses were done on the basis of the seven aforementioned domains. In the quantitative analysis the mean and standard deviation (SD) of each domain was computed. In the qualitative analysis the transcriptions related to each domain's items were analyzed using OpenCode software. The qualitative section complements the quantitative findings and additional quotes are provided to support them.

### Monitoring, supervision and quality control

The study protocol and both the English and Arabic versions of the tool were sent to the participating Arabic-speaking countries and the others received the English version only. The PI and the co-investigator were informed of the timings of the FGDs. Finally, the completed tools were checked with the transcripts, and occasionally the audio files, to assess the consistency of response.

### Ethical considerations

This study has been approved by the Institutional Review Board of Tehran University of Medical Sciences, which abides by the Helsinki Declaration. Verbal consent was taken from the participants because the study was not individual- based and it only explored the status of their universities/institutes regarding KT. Therefore, the study subjects were not under any procedure that could have harmed them. All participants had been provided with the study guide, which included an introduction to the objectives and the method of implementation of the study. Colleagues from the same university/institute witnessed the verbal consent taken from all participants. In addition, official letters of invitation were sent from the Research Policy and Cooperation (RPC) division in WHO/EMRO to participating universities/institutes. The names of the countries and institutes are not disclosed in the analysis of results to ensure confidentiality.

## Results

The research results are presented in two sections (quantitative and qualitative), which are considered complimentary. FGD participants were asked to provide quantitative as well as qualitative information/relevant statements, accordingly.

### Quantitative findings

Table 2 shows the mean scores and standard deviations for each of the participating institutes in the seven domains. It illustrates the quantitative results, where priority setting is the weakest domain in the region, being below a score of 2 in half the institutes, with only one institute scoring above average. The highest scores were obtained in the ‘Research quality & timeliness’ domain. The rest of the domains were more or less equal in strength and not in favorable states. In this table we have shown the means and SDs of the groups the countries fall in, in addition to their individual scores. The mean and SD of *each item* of the self-assessment tool for all the participating institutes are presented in table 3.

**Table 2:** Mean scores and standard deviations for each of the participating institutes in the seven domains†.

Domain	Priority setting		Researchers' knowledge translation capacities		Processes and regulations supporting knowledge translation		Interaction with research users		Facilities and pre-requisites of knowledge translation		Promoting and evaluating the use of evidence		Research quality & timeliness	
Institution‡	Mean	SD	Mean	SD	Mean	SD	Mean	SD	Mean	SD	Mean	SD	Mean	SD
**1.**	1.3	0.5	1.6	0.8	1.3	0.5	1.8	0.6	1.7	0.7	1.6	0.7	2.5	1.0
**2.**	1.5	0.6	2	0	2.4	1.6	2.4	1.2	2.7	1.1	2.3	1.4	2.4	1.3
**3.**	1.5	0.6	2	0.9	1.7	0.7	1.7	0.7	1.7	0.7	1.6	0.9	3.3	1.2
**4.**	1.5	0.6	1.8	0.8	2.2	1.3	2.5	1.4	2.7	1.5	2.3	1.1	2.7	1.3
**5.**	2.0	1.2	2.4	1.1	2.7	0.8	2.5	0.9	2.4	0.9	2.4	1.2	3.3	1.0
**6.**	2.1	0.6	3	0.4	2.7	1.0	2.9	0.8	3.0	0.9	2.7	1.6	3.9	0.7
**7.**	2.5	0.1	2.5	0.4	2.9	0.5	2.7	0.6	2.6	0.6	2.6	0.4	3.2	0.4
**8.**	3.6	0.5	3.5	1.1	3.5	1.1	3.2	1.0	2.9	0.9	3.0	0.8	4.4	0.8
**Group 1** ‡‡	0.9	0.9	0.8	0.8	0.9	0.9	0.9	0.9	0.9	0.9	1.4	1.4	0.9	0.9
**Group 2**	2.2	0.6	2.4	0.9	2.5	1.1	2.5	1.1	2.4	1.1	2.3	0.9	3.5	1.1
**Group 3**	1.8	0.5	2.0	0.5	2.2	1.0	2.3	0.8	2.3	0.8	2.2	0.9	2.7	1.0
**Overall scores**	2.0	0.9	2.3	1.0	2.4	1.2	2.5	1.0	2.5	1.0	2.5	1.1	3.2	1.1

† The sequence of domains has been arranged from minimum to maximum overall mean scores obtained (from left to right).

‡ For the sake of confidentiality, the names of the institutes were not disclosed, and their order in this table does not correspond to the countries in table 1.

‡‡ The classification of the countries is according to that illustrated in table 1, i.e. group 1, 2 and 3 are those with high income, middle income, and low income.

**Table 3:** The SATORI tool and the mean scores obtained per item in the 7 domains by all the participating institutes.

Domain/Item	Mean	SD
***Priority setting***		
**Regular meetings** are held for exchanging **research priorities** of individuals and/or research using organizations for identification of their **priorities**.	2.1	1.1
A website and/or data bank is available in our organization for conveying the **research priorities of other organizations**.	1.8	0.9
**Our organizations' research priorities** are determined through meetings with executive organizations' representatives and/or **users of research results** (like community representatives, patients etc.	2.2	0.8
**Our organizations' research priorities** are compiled and its **up-to-date** list is available to the organizations' researchers.	1.9	1.0
***Research quality & timeliness***		
Our impression is that the **users** of research results trust the **quality** of the research done in our organization.	4.0	0.7
**Quality assurance** program is required for each research (data gathering protocol and/or training the research workers).	3.2	1.7
**Quality control** is carried out while research is being conducted (internal monitoring of the executive program by the research group and/or external supervision).	2.9	0.9
The gap between ‘presentation of the research proposal’ and ‘beginning of the research’ is reasonable (the process of **reviewing the research proposal**).	3.3	0.9
While designing the research proposal and performing the projects researchers are aware that applied projects should reach results in good time (**the projects duration** and **absence of delay in performing them**).	3.8	0.7
The gap between ‘end of research’ and ‘finalization of results in the form of a report’ is reasonable (**the process of presentation of research results**).	3.4	0.9
**The gap between article submission and acceptance** in journals is such that the interventions that result from research can be implemented in reasonable time (considering the need for prompt availability of research results to decision makers.	1.9	0.8
***Researchers' knowledge translation capacities***		
Researchers are familiar with the topic of **knowledge translation** and how to perform it.	2.8	0.8
Our researchers convert their research results into **actionable messages** appropriate to the target audience.	2.3	0.8
Our researchers have **communication skills** for knowledge transfer.	2.6	0.6
Knowledge transfer and utilization of research results exist in the **general program of research methodology training**.	1.9	1.3
**A list of all the** **(**research result **users)** is prepared for each research project.	1.9	0.7
***Facilities and Pre-requisites of knowledge translation***		
Compared to the organization's internal budget for research, the amount of external funding is such that researchers **are encouraged to use external funding**.	3.3	1.3
In **research project proposals** (projects whose users are service providers, managers, policy makers, patient groups and/or people) **budget is considered for disseminating the results** (other than being published in peer-review journals and/or attending conferences).	2.2	0.7
Our researchers can use the **services** of those familiar with **knowledge transfer skills** (the presence of individuals in our organization who work with this objective; and/or make contracts with individuals and institutions outside our organization).	1.7	0.6
Our researchers have the necessary **financial resources** for preparing content appropriate to the target audience.	2.1	0.8
Our researchers have the necessary **equipment** for preparing content appropriate to the target audience.	2.7	1.0
Our researchers have adequate **time** for preparing content appropriate to the target audience.	2.7	0.9
**The necessary structure** (like office and/or organizational unit) and/or **manpower** is available for strengthening knowledge transfer in our organization, considering the produced amount of research-based knowledge transferable to the decision makers.	2.3	1.4
Our organizations' research managers are aware of the researchers **needs** (separately for each study field-group etc) in the field of knowledge transfer, and perform proper interventions for them.	2.4	0.8
Researchers can provide the results of their research through the **web** and/or **electronic banks**.	2.7	1.1
***Processes and regulations supporting knowledge translation***		
Researchers are **motivated to use external funding**. (the extra-organizational part of the process is easier).	2.6	1.7
In case of **external funding**, researchers can use these for research matters **easily and in a short period of time**. (the intra-organizational part of the process).	3.0	1.2
**Our organization provides researchers** with **incentives to use external funding.**	2.6	1.2
Research studies that result in **production** of ‘**actionable messages**’ with a high level of evidence (such as regular systematic reviews and/or clinical guideline development activities) are considered priorities of research and granted funds.	2.8	1.0
In our organization there is a process that determines **which research** results can be transferred (keeping in mind the fact that not every research result is transferable) to the target audiences (‘other than’ transferring to other researchers and funders).	2.4	0.7
In our organization, all research results are **peer reviewed** prior to knowledge dissemination or transfer.	2.8	1.4
**Our researchers** have **the necessary incentives** for performing knowledge transfer (rewards, appropriate promotion rules).	1.7	0.9
**Intellectual property rights** exist which support researchers who help disseminate research results prior to their publication in journals.	2.1	1.3
**There are criteria for evaluation** of researchers' knowledge transfer activities in our organization.	1.7	0.7
Our organization has **regular communications with public and private media** and target audiences (like publications related to women and youth) for transfer of research-based evidence.	2.4	1.2
The format of **peer review journals** which publish research results is such that the decision makers are easily informed of the **actionable message** when necessary.	2.4	1.2
The framework of research projects' **final reports** are such that decision makers can easily point out the actionable message.	2.5	1.2
***Interaction with research users***		
In our organization there is a comprehensive **list** of **organizations** that can use our research results.	2.4	0.9
The **particulars** of each unit's **researchers** and their capabilities are made available to other organizations through a **databank**.	2.0	0.9
Individuals and decision-maker organizations know which fields **our organizations' research capacities** cover.	3.2	0.7
For preparing grounds for performing relevant research and strengthening research utilization, our organization holds **regular** and **purposeful meetings** with decision-makers (managers and policy makers) for **extending cooperation** and using **mutual capacities** (establishment of a knowledge network).	2.2	1.0
The groups which will use the results of research **participate** in its design and/or conduct.	2.4	1.0
**Meetings** are held for **presentation of research results** to decision makers.	3.0	1.2
***Promoting and evaluating the use of evidence***		
**Evidence-based decision making** (based on domestic and/or foreign research)is among the subjects of research in our organization.	2.9	0.6
Our researchers study the extent to which **decision makers utilize** our organizations' **research results**.	1.4	0.5
Our researchers identify the potential **barriers of behavioral change in decision makers** for utilizing their research results.	1.8	0.8
We conduct education programs such as ‘**evidence-based medicine**’ or ‘**evidence-based decision making**’ for service providers and/or managers.	3.1	0.9
Systematic reviews and clinical guidelines…etc that strengthen evidence-based decision making are produced in our organization.	2.2	1.1
Our researchers play an active role in technical committees that help in decision making (executive organizations' decision making, hospital management and also groups supporting the health of patients and people).	3.4	1.1
We send decision makers **reminders** to follow the research results that we've previously sent them.	1.5	0.7

### Qualitative findings

With the exception of one country, each held 2 FGDs.

The qualitative findings have been explained according to the domains specified. The participants' quotes have been stated where felt necessary. To better clarify the subject, a short explanation is given at the beginning of each domain.

### Priority setting

Priority setting refers to whether research is regularly chosen on the basis of research user organizations' needs or opinions or not. In our study, we found that priorities are in many cases set by the Ministry of Health (MoH). However in many instances, they are determined on individual basis, defined by the clients or on the basis of national or organizational needs, e.g. hospital-related issues. Hence, regular meetings are not usually held with research end-users for priority settings; in fact most of the countries did not hold any meetings for this purpose. There were however instances of priority setting through meetings with stakeholders such as non-governmental organizations, municipality and patients.

*“We do not routinely have a platform to conduct meetings for other organizations/end users of our research”.*

Most of the institutes had websites, but they lacked specific databases of their own or other organizations' priorities. Even if such websites existed, their databases were not up-to-date.

### Research quality and timeliness

The quality of research has a direct relationship with KT, because the higher the quality of evidence produced the greater the value of the knowledge transferred. In this regard, the participants believed that most end-users trusted the quality of their research results. Their opinions on quality assurance and quality control were different though. Mostly they believed that the status of these two were variable and depended on the amount of budget and whether the projects were internally or externally funded, reflecting the notion that the source of funding affects the quality management of the projects.

In addition to quality, timeliness in conducting research also affects KT. Bearing in mind the pace with which scientific developments grow, delays in KT may undermine the value of the evidence or make it un-usable. In this context, we found that the reported time for proposal review was variable depending on the type of research and funding. And even though most of the researchers were aware of the need for timeliness in conducting research, this known fact did not prevent delays, and only one participating institute was satisfied with the timing in its research. The time taken to present research results also varied according to the size of the research, and whether it was an undergraduate or graduate research project. Finally, the gap between article submission and acceptance in journals was not desirable in most cases. According to the participants, if the MoH was to wait for the research results to be published, then certain interventions would not be carried out in the appropriate time to be effective.

*“It takes 6 months to 3 years to publish 1 simple article… this is my experience… it is very difficult”.*

### Researchers' knowledge translation capacities

Capacity building is required to enable researchers to work in the field of KT. However, according to our findings, only one institute had included KT among the topics taught in research methodology courses. Hence, it came as no surprise when we learnt that more than half the participants believed that researchers were not familiar with KT. Naturally, when researchers are not familiar with KT, they will not be able to convert their research results into actionable messages. Only one institute believed it had this capability. Now even if the researcher has this capability s/he must know who to deliver the message to. However, a clear list of research users was only sometimes available to the researchers. This action too requires certain communication skills. Most participants believed that researchers did possess communication skills for KT, but whether they used them or not was another issue.

*“There are certain recommendations, but they're less appropriate for use in reality.”*

### Facilities and pre-requisites of knowledge translation

By ‘facilities and pre-requisites’ we are referring to any tangible equipment, facility or provision that facilitates or is necessary for performing KT activities. One of the appropriate structures for strengthening KT is in the form of a KT office that works under the supervision of the Directorate of Research of that institute/university. Such an office can support or guide researchers in their KT endeavors and promote the uptake of research findings across the country, by helping throughout the process of conducting research. Only one institute had recently run such an office. However, most of the participating institutes did not have this facility. Another was in the process of setting it up. Moreover, almost half the participating institutes did not have the necessary infrastructure for preparing and displaying KT contents. The others had no problem with the infrastructure, but whether they used those facilities or not was another issue.

Money, manpower and time are considered pre-requisites of KT activities. Almost half the participating institutes did not have financial support for KT activities. One institute mentioned that they were in the process of allocating this budget, while the others said that acquiring the budget was not a problem, but that they never asked for this budget in the first place or that human resources were scarce.

More than half the participating institutes mentioned that their staff did not have time for preparing KT content and none could use the services of individuals familiar with KT skills either. And although research managers were aware of researchers' KT needs in more than half the cases, they did not take the necessary steps to fulfill their needs.

Many institutes are encouraged to use external funding for research because of its magnitude, and therefore meet their clients' needs. In some institutes this type of funding is the only one available. In others, although some were encouraged to take up their national interests and conduct research for their MoH, they complained that there was lack of proper funding for identified research priorities by the MoH.

*“MoH should increase its contribution to research funding.”*

*“When the MoH suggests topics for research, it does not state that they are funded ones.”*

### Processes and regulations supporting knowledge translation

This domain covers the bureaucratic processes and regulations supporting and encouraging KT. Hence, all the items listed in this domain support KT in one way or another, either by encouraging the conduction of client-oriented research, or fostering the production and delivery of actionable messages. Knowledge-generating research can support decision making by producing actionable messages. Among these are systematic reviews and clinical practice guidelines. In our study, we found that in comparison to other types of research, these types of research did not receive much support in most cases and the researchers thought they were too time-consuming.

*“The applicable theses are only devoted to engineering and agricultural researches as they consider their end-products marketable, but medical ones are considered to be inapplicable, so research in the health field is neglected.”*

There is no clear and well-defined process determining which research results should be transferred to the target audience. Only in one case, it was being shaped. In half of the institutes regular communications with the media was mainly for the purpose of transferring research results.

One of the ways of strengthening KT is conducting client-oriented research in which the research is directly addressed to the user. However, securing external grants was not favored in most cases; where mostly there were bureaucratic hurdles on both sides. The extra-organizational part of the process is very long and tiresome, and researchers are not usually enthused to use this option, because the mechanisms are not easy. Some did believe they were encouraged but it depended on the donor organizations. Even the intra-organizational part of the process was considered more difficult by half the participants. The other half thought it was easier; though one institute mentioned that in case of foreign funding, it was more difficult (i.e. easier with domestic funding).

*“There are many overhead costs involved- which are the negative aspects.”*

Research results need to be peer reviewed prior to dissemination in order to present the users with correct and reliable information. According to our findings, peer review was not performed in most cases. On the other hand, the formats of journals and final reports can facilitate the transfer of knowledge by presenting actionable messages, readily available to those who are looking for lessons learnt and guidelines for decision making. Only one institute stated having introduced this type of format in one of its peer reviewed journals, and is working on its development in other journals and final reports as well. Another institute stated not having any peer reviewed journals at all.

*“Most reports include visionary statements and concrete concepts are not given. The actionable messages given are mostly broad statements and not really actionable.”*

Moreover, researchers' intellectual property rights need to be safeguarded too. If a researcher is asked to disseminate her/his research findings before they are published s/he needs to be sure that her/his intellectual rights are not violated. In our study however, only a few believed that such rights existed. Another institute believed that patents were not well supported.

KT evaluation criteria were absent in most of the institutes in our study, though a few believed they were “beginning to develop them” or “existed but were weak”. Even if there was a proper evaluation, most participating institutes did not have incentives for performing KT activities. Only one institute stated that their MoH provided bonuses to research projects which have applicable results.

*“When there is no transfer there is no evaluation either!”*

*“There are no monetary incentives like upgrading”.*

### Interaction with research users

One of the necessary elements of KT is having interaction with research users; in this case ‘decision makers’ [22], [23]. Certain requirements are there to fulfill this purpose. Among them is a list of organizations that can use research-producing-organizations' research results. According to the participants, this was not available in a comprehensive or tangible form. Their organizations' research capacities were actually known to decision makers in only a few cases. Moreover, there was no discrete databank of researchers' particulars available. Knowledge networks as such were present in a few cases only, but they were not very active or functional. In other cases, there were no knowledge networks, neither formal nor informal, but meetings were occasionally held to decide on research priorities and topics. Meetings were sometimes held for presentation of research results to decision makers, but not on a regular basis.

*“We are doing things but in fragments and mostly at macro level.”*

*“The end users do not know where to seek information from us either.”*

*“… strong collaborative ties should be developed between all the concerned bodies in order to make the most out of scanty funds and available resources.”*

### Promoting and evaluating the use of evidence

This domain deals with promoting EBDM through various steps, its training and development of knowledge products (such as systematic reviews and clinical guidelines), and whether such measures result in behavior change in decision making or not.

In this regard, systematic reviews were only produced in certain institutes, and some mentioned that since international guidelines are available, local ones are not designed. Moreover, the financial barrier was mentioned in one case:

*“Systematic review productions are among the university's priorities, but it does not allocate sufficient funds to them.”*

However, the pre-requisite of using evidence in decision making is to receive the necessary training in this field. Only half of the participating institutes conducted regular EBDM training programs for both decision makers and researchers; the remaining either did not hold such programs or conducted them only for service providers, and not for decision makers. In fact, they believed decision makers were not very interested in attending such workshops. On the other hand, researchers' roles as knowledge producers cannot be overlooked in the decision making process. According to our findings, the researchers of only one institute did not attend technical committees that help in decision-making organizations. However, they did not send reminders to decision makers to follow uptake of their evidence.

*“We don't even send the original research results, let alone reminders!”*

*“Basically we send nothing.”*

Even when the research results are published or sent to potential users, the extent to which those results are applied to decision-making is not followed-up. Nor do they identify the potential barriers of behavioral change at decision-making level. Others believed that the high turn-over of officials at the MoH was itself an obstacle to behavioral change, because by the time a manager is trained or decides to carry out a certain intervention s/he is replaced with another. Naturally, EBDM was not included in their research studies either, because the uptake of evidence was not an issue in the first place.

*“Once published, we do not follow.”*

*“After the research is over most researchers seek promotion and rewards but are not concerned with how much people benefit from it”.*

*“There is no proper follow-up and/or collaboration with the Medical Council on how to enforce these guidelines and ensure their implementation.”*

*“One writes a report and then forgets about it”.*

## Discussion

This study was done with the purpose of identifying the strengths and weaknesses of KT in selected EMR countries/institutes by using the SATORI tool, through qualitative and quantitative methods. According to the results of this study, in spite of the research institutes' strengths in producing quality research, they face shortcomings in applying this knowledge. There are weaknesses in capacity building of researchers, processes and regulations supporting KT, facilities and pre-requisites of KT, interaction with research users and strengthening of EBDM. However, the quantitative and qualitative findings of the study both show that the institutes under study have an undesirable status regarding priority setting in line with health priorities and decision makers' needs. Hence, this domain requires more effective interventions.

In 1990 the ‘Commission on Health Research for Development’ reported that only 10% of the budget allocated to health research was spent on health problems of 90% of the world's population; what was later coined by the ‘Global Forum for Health Research’ as the ‘10/90 gap’ [24], [25]. Priority setting was therefore recognized as a priority that needed strengthening in the health arena [5]. The ‘Council on Health Research for Development’ then developed a tool by the name of ‘combined approach matrix’ to help set priorities of health research to support health, development, and equity by involving the main stakeholders of health and health research [25]–[27].

In spite of these efforts, it seems that priority setting is not considered a priority in low and middle-income countries; other studies conducted in the region also confirm our findings [15], [17]. Since the budget allocated to research is generally low in these countries [5], it is even more important to conduct research on the basis of national or local priorities.

One reason research is not performed on the basis of health priorities may be the gap between evidence-producing institutes (particularly academic ones) and decision makers in the health system; a barrier that has been met by establishing knowledge networks [28]–[30].

There are problems in the other domains as well, even though their scores are relatively better. Researchers' familiarity with KT issues and their KT skills are not noteworthy. The region needs to work on the universities and institutes' KT capacity building and empower researchers to increase their capacities in communicating with stakeholders and translating their findings. Educational programs can be effective in this regard. Moreover, researchers and decision makers alike may be enabled to use the services of individuals or organizations as knowledge brokers. In fact, knowledge brokers can help link researchers to users [31]–[34].

Similarly, in the ‘Processes and regulations supporting knowledge translation’ domain only one participating institute scored above the threshold. For example, inclusion of KT activities in promotion criteria, observing intellectual property rights, and granting awards to the benefit of KT exist in pioneer countries. Including such regulations can encourage researchers toward KT activities [35], [36].

Researchers’ ‘interactions with the end users’ of research was not adequate in the institutes we studied. El-Jardali et al studied researchers' opinions and experiences on the role of research evidence in health policy making in EMR countries. They found that researchers' interactions with end users of research were insufficient, although some health researchers did recognize the importance of interaction with policy makers [15].

The ‘Facilities and pre-requisites of KT’ scores show that KT is not a very costly measure, implying that research organizations are not concerned with the concept of KT in the first place.

There is apparently no problem with the quality of research. Almost all participating institutes have scored relatively well. This may be due to three reasons. Firstly, they were among the best institutes within their own countries, which hint to possible ‘selection bias’. However, we purposefully searched for institutes that had a high rate of knowledge production to be able to assess their KT activities. Secondly, it might be a kind of ‘information bias’, since most participants were researchers. Thirdly, the participants were not aware of problems in timeliness of research. In any case, this domain does not appear to be an urgent demand for intervention.

Other limitations included finding focal points which was extremely difficult. Moreover, the response rate was not good. Therefore, the samples in this study are not necessarily representative of the entire region [37], [38]. Moreover, since only one institute was selected from each country, the status of one institute is not necessarily representative of the country's status.

In this study the sampling was such that samples were selected from each of the region's three groups- based on the status of their health systems. The point worth noting is that, according to table 1, the statuses of all the indicators of group 2 countries were better than the other two groups. The status of group 3 countries was even better than group 1 countries, indicating the need to further develop research infrastructures in countries of higher economic status [17].

Furthermore, the results of the study may be based on the impression of the study participants. In other words, the institutes cannot be ‘compared’ with each other, because the desirable level of the participants may differ from each other. The comparisons are significant only for the values within an organization. Moreover, the identified weaknesses are not necessarily ‘needs’, but rather ‘demands’. However, the demands perceived by the decision makers in the health research system can help bring about change in the system.

One of the advantages of using the SATORI instrument for the KT assessment is the use of qualitative data to support quantitative data which strengthens the validity of results, in general. Moreover, by sharing the draft of the results with all focal points and taking their comments into consideration, the trustworthiness of the findings increases. Furthermore, the discussions held in the process yield useful recommendations for changing the status of KT in the institutes.

Up to now, most KT studies have focused on the ‘uptake’ of evidence in decision making, i.e. ‘pull’ activities in KT [15]. On the other hand, to our knowledge, fewer studies have been conducted on how much knowledge is produced in the research sector and whether it is transferred to decision makers and relevant stakeholders, i.e. the ‘push’ side of KT. It could therefore shed light on this aspect of KT.

Upon further consideration of the state of health research in EMR countries the following points come to light. Firstly, it is clear that EMR countries are heterogeneous with respect to their status regarding research. By considering publications as a product of research, some countries do not have any publications at all, while some have over five thousand publications in the field of medicine alone [39].

At the same time, most of the countries in the region have fragmented health research systems, and the links between their components are weak for formulating the research question and eventually producing and utilizing its research findings. The research sector is therefore unable to meet the country's health and equity needs [17].

The fact that there are shortcomings in almost all studied domains, particularly in priority setting, leads to the conclusion that planning for and supporting KT does not happen at the macro level in the health research system in the first place. The research systems do not seem to be concerned with addressing the health systems' needs, in spite of the steps taken towards incorporating and acculturating KT in the region by some countries [37], [38], [40]. In conclusion, it seems that the main problem is that a KT climate does not exist in the region. However, despite the difference in the contexts, there are many similarities in the region's institutes. International medical/health organizations, such as WHO, can play a role in supporting this climate by steering countries towards KT and suggesting regional strategic directions according to their needs.
