# Correction: The Knowledge Translation Status in Selected Eastern-Mediterranean Universities and Research Institutes

**DOI:** 10.1371/journal.pone.0130916

**Published:** 2015-06-12

**Authors:** Katayoun Maleki, Randah R. Hamadeh, Jaleh Gholami, Ahmed Mandil, Saima Hamid, Zahid Ahmad Butt, Abdulaziz Bin Saeed, Dalia Y. M. El Kheir, Mohammed Saleem, Sahar Maqsoud, Najibullah Safi, Ban A. Abdul-Majeed, Reza Majdzadeh

The affiliation for the fourth author is improperly abbreviated. The correct affiliation for Ahmed Mandil is: King Saud University–College of Medicine, Riyadh, Kingdom of Saudi Arabia.
