# Correction: The Knowledge Translation Status in Selected Eastern-Mediterranean Universities and Research Institutes

**DOI:** 10.1371/journal.pone.0221844

**Published:** 2019-08-26

**Authors:** Katayoun Maleki, Randah R. Hamadeh, Jaleh Gholami, Ahmed Mandil, Saima Hamid, Zahid Ahmad Butt, Abdulaziz Bin Saeed, Dalia Y. M. El Kheir, Mohammed Saleem, Sahar Maqsoud, Najibullah Safi, Ban A. Abdul-Majeed, Reza Majdzadeh

The affiliation for the seventh author is improperly abbreviated. The correct affiliation for Abdulaziz Bin Saeed is: Department of Family and Community Medicine, College of Medicine, King Saud University, Riyadh, Saudi Arabia.
